# Supplementary material for: The mechanism of variability in transcription start site selection
Source: eLife. 2017 Nov 23;6:e32038. doi: 10.7554/eLife.32038 (PMC5730371; doi:10.7554/eLife.32038)
Supplement: Supplementary file 1. [file elife-32038-supp1.doc]

Supplementary File 1. Oligonucleotides

| **name** | **description** | **sequence (5' to 3')** |
| --- | --- | --- |
| JW 30 | mutagenesis primer for introduction of β'D460A mutation | GGCATATAACGCCGCGTTCGATGGTGACC |
| JW 61 | primer for for primer-extension mapping of TSS and β'R1148-Bpa crosslinks to nontemplate strand of *lac*CONS | CGGCATCACCATCGGCATTGAC |
| JW 62 | primer for primer-extension mapping of β'T48-Bpa crosslinks to template strand of *lac*CONS | CTCCAGGTACCCGCAATAAATGTG |
| JW 85 | forward primer for amplification of *lac*CONS-GGG and *lac*CONS-CCT templates and mapping of β'T48-Bpa crosslinks to template strand | GTTCAGAGTTCTACAGTCCGACGATC |
| S128a | reverse primer for amplification of *lac*CONS-GGG and *lac*CONS-CCT templates and mapping of β'R1148-Bpa crosslinks to nontemplate strand | CCTTGGCACCCGAGAATTCCA |
| S1219 | forward primer for amplification of *lac*CONS*-*GGG and *lac*CONS-CCT templates | TATAATGCCTGACCGGCGTTCAGAGTTCTACAGTCCGACGATC |
| S1220 | reverse primer for amplification of *lac*CONS*-*GGG and *lac*CONS-CCT templates | AATTAAGCCGCTGGGGCCCTTGGCACCCGAGAATTCC |
| LY10 | forward primer for amplification of *lac*CONS template | GAGAGTTCTAGATCCAGCCTGCGGCCCAGAG |
| LY11 | reverse primer for amplification of *lac*CONS template | GAGAGACCTGCAGGGCCCGGATCCAGATGCTCTCC |
| Taq_rpoC_F | forward primer for amplification of 2 kb fragment of *T. aquaticus* *rpoC* to generate pUC18-T20C2 | GAGAGATCTAGAGACCTTCTGGATCTCGTCCACCAGG |
| Taq_rpoC_R | reverse primer for amplification of 2 kb fragment of *T. aquaticus* *rpoC* to generate pUC18-T20C2 | GAGAGACCTGCAGG ACATCAAGGACGAGGTGTGG |
| XbaRPOC4050 | primer for amplification of biotin-containing 1 kb fragment used in nanomanipulation experiments | GAGAGTTCTAGAGACCTTCTGGATCTCGTCCACCAGG |
| RPOC3140 | primer for amplification of biotin-containing 1 kb fragment used in nanomanipulation experiments | CTGATGCAAAAGCCCTCGGG |
| SbfRPOC50 | primer for amplification of digoxigenin-containing 1 kb fragment used in nanomanipulation experiments | GAGAGACCTGCAGGGAGAAGATCCGCTCCTGGAGCTACG |
| RPOC820 | primer for amplification of digoxigenin-containing 1 kb fragment used in nanomanipulation experiments | TCCTGGCGCAGGTAGATGAG |
